# Recalled Maternal Rearing Behaviour of Individuals Born Preterm and Their Mothers: The Impact of Intimate Mother–Child Contact

**DOI:** 10.3390/jcm13061822

**Published:** 2024-03-21

**Authors:** Daniëlle Otten, Mareike Ernst, Alexander K. Schuster, Jonas Tesarz, Sandra Gißler, Eva Mildenberger, Norbert Pfeiffer, Manfred E. Beutel, Achim Fieß

**Affiliations:** 1 Department of Psychosomatic Medicine and Psychotherapy, University Medical Center of the Johannes Gutenberg, University Mainz, Langenbeckstraße 1, 55131 Mainz, Germany; 2 Department of Child and Adolescent Psychiatry and Psychotherapy, University Hospital Ulm, 89075 Ulm, Germany; 3 Department of Clinical Psychology, Psychotherapy and Psychoanalysis, Institute of Psychology, University of Klagenfurt, 9020 Klagenfurt am Wörthersee, Austria; 4 Department of Ophthalmology, University Medical Center of the Johannes Gutenberg, University Mainz, 55131 Mainz, Germany; 5 Department of General Internal Medicine and Psychosomatics, University Hospital Heidelberg, Heidelberg University, 69117 Heidelberg, Germany; 6 Division of Neonatology, Department of Pediatrics, University Medical Center of the Johannes Gutenberg, University Mainz, 55131 Mainz, Germany

**Keywords:** prematurity, preterm, parenting, parental behaviour, mental health

## Abstract

**Background**: Preterm birth is a risk factor for a variety of detrimental health outcomes. Previous studies have identified recalled (or remembered) parental rearing behaviour as a potential modifier of preterm individuals’ mental health in adulthood. However, no investigations to date have contrasted the parents’ and children’s views, explored whether their congruence is associated with preterm individuals’ mental health, or tested associations with maternal self-reported first skin-on-skin contact. **Methods**: This cohort study involved 199 participants of the Gutenberg Prematurity Eye Study (GPES), with prospective clinical examination and psychological assessment data available for individuals born preterm and term and their mothers’ perspective on recalled parental rearing behaviour. Participants also completed the Patient Health Questionnaire-9 (PHQ-9). **Results**: There were substantial similarities between reported recalled maternal rearing behaviour of individuals born preterm and at term and their mothers, with individuals born preterm with lower gestational age (age of the pregnancy from the woman’s last menstrual period) recalling mothers as comparatively more controlling and overprotective. Incongruence in recalled rejection/punishment was associated with more depressive symptoms. Late first skin-to-skin contact was related to more recalled maternal rejection/punishment, less emotional warmth, and more control/overprotection. **Conclusions**: this study expands the knowledge about the interrelations of preterm birth, maternal rearing behaviour, and mental health, underscoring the relevance of first relationship experiences, including close intimate contact.

## 1. Introduction

Preterm birth, occurring before the completion of 37 weeks of gestation, accounts for approximately 11% of global births [1], with rates rising in many countries around the world [2]. However, the preterm birth rate has remained stable at 8% since 2008 in Germany [3]. Preterm birth has several negative consequences for its survivors and, while medical progress has significantly reduced the mortality rate for infants born preterm in recent decades, preterm birth continues to affect the physical and mental health, cognitive and social development, and well-being of survivors in adult life [1,4,5,6].

For parents, a child born preterm involves (intense) treatment protocols, frequent hospital visits, and worries about their child’s health and prognosis. A meta-analysis revealed slightly more mental distress in parents of children born preterm compared with parents of term-born children [7], especially when the child had a low gestational age (GA) and birth weight. Parents of preterm children were particularly burdened when they had to take considerable time off work, had financial worries and increased debt, and experienced unsafe home environments and social isolation [8]. The effects of preterm birth seemed to be especially strong for mothers, as they reported more distress than fathers at birth [7], more postnatal health problems [9], and displayed a higher prevalence of post-traumatic stress [10,11].

Preterm birth and the subsequent parental distress may affect child-rearing behaviour. The empirical literature has shown differences in child-rearing behaviour between parents of vulnerable children (children with chronic illnesses or life threats but also those born preterm) and non-vulnerable children [12]. Child-rearing practices can be divided into “care factors” such as acceptance and warmth versus rejection and criticism, and “control factors” such as parental control and overprotection versus promotion of autonomy. Parental stress is higher among parents of children with intellectual (e.g., Down syndrome, autism) [13] and developmental disabilities [14] compared with parents of children without disabilities. Parental demandingness has been linked to enhanced cognitive development in children, while the levels of parental warmth and experiences of rejection are correlated with behavioural outcomes in children. In children born very preterm (fewer than 33 completed weeks of gestation), positive associations between difficult temperament in infancy and negative affectivity in childhood occurred regardless of parental rearing behaviour [15].

Mothers were shown to have less contact with their babies born preterm, with less positive feelings towards them [9]; such alterations in the early caregiving environment could affect infant attachment. Although a recent study did not find differences in attachment styles between infants born preterm and at term who were studied 9 months postnatally [16], other studies revealed associations between intimate mother–child contact after birth and attachment styles and parental rearing behaviour in primary school children (mean age of 7). For example, breastfeeding might contribute to a child’s secure attachment in its first and second year of life [17]. Parental rearing behaviour has been associated with the development of specific attachment styles in children; for instance, children with an ambivalent attachment style often view their mothers as overly protective, anxious, and lacking in warmth, whereas those with an avoidant attachment style tend to see their mothers as less warm and protective [18].

Current animal studies have shown that separation from the mother leads to toxic stress in the baby’s brain [19]. High cortisol levels due to separation can cause long-term damage. A study on rats showed that rats that received less attention, care, and grooming from their mothers had a different way of dealing with cortisol. In rats with less maternal care, cortisol takes much longer to be removed from the bloodstream and the production of cortisol continues because the negative feedback loop is weaker [20,21]. Moreover, a human study demonstrated that infants who slept without skin-to-skin contact with their mothers experienced an 86% reduction in quiet sleep and a 176% rise in autonomic activity compared with infants who had skin-to-skin contact [22].

An important form of intimate mother–child contact is skin-to-skin care (so-called kangarooing). Kangarooing not only has positive effects on an infant’s neonatal physiological parameters [23], perceptual and cognitive development [24], stress regulation [25], and motor development [24] but also on the parenting process [24], in particular bonding [23]. Early maternal touch may affect an infant’s socioemotional development and attachment quality, which has positive implications for mother–child relationship functioning [26]. However, the long-term effects in adulthood are still unclear.

Reflecting on this context, our study adopts a retrospective approach, exploring the long-term consequences of preterm birth on parental rearing behaviours and the mental health of individuals born preterm. “Recalled” parental rearing behaviour and its influence on adult mental health outcomes form the core of our investigation. This methodological choice underlines the importance of understanding how past experiences, as remembered by participants, shape present realities for families affected by preterm birth.

Studies comparing retrospective views of parents and their children on rearing behaviour are scarce. Some investigations report substantial disagreement between reports of children and parents [27], but implications regarding the child’s mental health as well as the parent–child relationship are inconclusive [28,29]. To our knowledge, no such studies are available in the context of prematurity, which is of particular importance because our previous report demonstrated that prematurity influences rearing behaviour, showing an association with children’s mental health in adulthood [30]. Thus, the question arises of how far the reports of individuals born preterm are congruent with those of their mothers and whether potential discrepancies in recalled rearing behaviour between individuals and their mothers have implications for well-being. Therefore, this study aimed to compare the recalled maternal rearing behaviour of mothers and their adult children to shed light on the interrelations of intimate perinatal mother–child contact and recalled maternal rearing behaviour in adulthood. This study also examined whether discrepancies in recalled maternal rearing behaviour between individuals (preterm and term-born) and their mothers affect individuals’ mental health. The research questions aim to investigate this relationship while considering that any observed differences between preterm and term-born individuals could stem from a variety of factors. By doing so, we do not investigate direct causality from prematurity alone, thus acknowledging the complexity and multifaceted nature of these relationships. The research questions are as follows:

(1) To what extent is recalled maternal rearing behaviour from the perspective of adults born preterm and their mothers congruent?
(2) Does early intimate mother–child contact (skin-to-skin contact, breastfeeding, and proximity at night) positively affect recalled maternal rearing behaviour decades later?
(3) Is the incongruence in recalled maternal rearing behaviour between individuals born preterm and at term and their mothers associated with depressive symptoms in adulthood?

## 2. Materials and Methods

### 2.1. Study Population

The Gutenberg Prematurity Eye Study (GPES), conducted at the University Medical Center of the Johannes Gutenberg-University Mainz (UMCM) in Germany, is a single-centre retrospective cohort study complemented by prospective clinical and psychological evaluations [31]. Between 2019 and 2021, the study selectively recruited participants by inviting every second preterm newborn (born with a gestational age (GA) of 33−36 weeks) and all preterm newborns (with a GA ≤ 32 weeks) delivered at UMCM from 1969 to 2002 who were aged between 18 and 52 years at the time of study participation. To establish a control group, for each birth month spanning from 1969 to 2002, six term-born individuals (three males and three females) within the 10th to 90th birth weight percentiles were randomly selected and invited to participate as controls [32,33,34,35]. Of the 40,189 selected individuals, *N* = 938 could be contacted, and *N* = 450 formerly premature individuals agreed to participate in the study.

Only participants who completed the parental rearing behaviour questionnaire and whose mothers completed the maternal rearing behaviour questionnaire were included in this study, (*N* = 199). The sample comprised 64 participants born at term, 61 participants born at a GA between 33 and 36 weeks, 47 participants born at a GA between 29 and 32 weeks, and 27 individuals born at a GA equal to or less than 28 weeks. All participants provided written informed consent and all GPES procedures complied with good clinical practice (GCP), good epidemiological practice (GEP), and the ethical principles of the Declaration of Helsinki. The study protocol and study documents were approved by the local ethics committee of the Medical Chamber of Rhineland-Palatinate, Germany (reference no. 2019-14161).

### 2.2. Data Collection

All participants provided current sociodemographic and clinical information via self-report questionnaires and a clinical interview with a physical examination, during which questionnaire data were validated and missing information was added.

### 2.3. Assessment of Sociodemographic Characteristics

Data were collected about participants’ gender, age, working status, marital status, and socioeconomic status (SES), which was defined according to Lampert et al. [36]. The metric index ranged from 3 (lowest SES) to 21 (highest SES) and combined information on education, occupation, and income with equal weights. SES was categorised into three groups: low (<9), moderate (9–15), and high (16–21) SES.

### 2.4. Assessment of Pre-, Peri-, and Postnatal History

The medical history of participants was evaluated using their medical records held at the University Medical Center of the Johannes Gutenberg-University Mainz (UMCM). Key variables of interest included gestational age (GA) (measured in weeks) and birth weight (recorded in kilograms). For this study, birth weight percentiles were determined based on the methodology described by Voigt et al. [37], mother’s age at birth, maternal smoking and alcohol consumption during pregnancy, pre-eclampsia, need for intubation, and perinatal adverse events. Perinatal adverse events were defined in congruence with the German query for quality control of neonatal clinics [38] as an occurrence of intraventricular haemorrhage (at least grade 3 or parenchymal haemorrhage) and/or necrotising enterocolitis and/or moderate or severe bronchopulmonary dysplasia. Information about the postnatal clinical course included time on invasive ventilation (in days), time spent in an incubator (in days), time spent in the intensive care unit (ICU) (in days), and length of stay in the hospital (in days). Perinatal medical charts were utilized to document umbilical artery pH, Apgar scores, and the highest recorded pCO_2_ levels during the hospital stay. The Apgar score, a 10-point scale assessing newborn health across 5 criteria (respiratory effort, heart rate, reflex irritability, muscle tone, and skin coloration), is conducted right after birth. It serves as a widely recognized and straightforward tool for evaluating a newborn’s immediate health status post-delivery. Lower Apgar scores can signal the need for additional care, including respiratory support [39,40,41].

### 2.5. Assessment of Rearing Characteristics

Recalled rearing behaviour was assessed using the 12-item version of the Recalled Parental Rearing Behaviour Questionnaire [42] (originally derived from the Swedish scale “Egna Minnen av Barndoms Uppfostran (EMBU)” (English: “My memories of upbringing”) [43]. Only the recalled rearing behaviour of mothers was included in this study since too few fathers filled out the questionnaire. Six items describe experiences referring to rearing style and specific behaviour, e.g., “Did your mother comfort you when you were sad?” (assessing emotional warmth), and were summarised under three scales: emotional warmth, rejection/punishment, and control/overprotection. Participants were asked to judge the frequency of the respective behaviour on a Likert scale ranging from 0 = “no, never” to 3 = “yes, always”. The full recalled parental rearing behaviour scale has previously shown good psychometric properties and measurement invariance concerning gender and age [44,45,46]. The subscales showed satisfying to good internal consistency ω = 0.82 (emotional warmth), ω = 0.76 (rejection/punishment), and ω = 0.71 (control/overprotection) [46]. In line with a previous study on recalled parental rearing behaviour in individuals born preterm, we investigated the two items of the Recalled Parental Rearing Behaviour Questionnaire’s control/overprotection scale not only as a sum score but also separately [30]. Furthermore, these two dimensions were referred to as parental ambition and overprotection, as this was more in line with their contents. Therefore, the present study investigated five dimensions of parental rearing behaviour: emotional warmth, rejection/punishment, control/overprotection, ambition, and overprotection.

### 2.6. Assessment of Intimate Mother-Child Contact

Information on early intimate mother–child contact was extracted from the self-report questionnaires and clinical interviews of the mothers of preterm infants and was therefore also based on recalled information. Skin-to-skin contact was measured in relation to what day after birth mothers could cuddle with their newborn or hold their newborn on their chest. Breastfeeding was directly measured by asking whether mothers breastfed their newborns or not. Proximity at night was measured with the question, “From what day after birth mothers could sleep together with their newborn (so-called rooming-in)?” The reliance on recalled information from years ago presents challenges, potentially leading to memory biases in our results. Although there are methods to adjust for and reduce these biases, applying them in practice can be intricate and is limited by available resources.

### 2.7. Assessment of Depression

The Patient Health Questionnaire’s depression module (PHQ-9) requires participants to report how often they have experienced symptoms aligned with the nine diagnostic criteria for major depression in the last two weeks, with responses ranging from 0 (“not at all”) to 3 (“nearly every day”). The PHQ-9’s total score, which can vary from 0 to 27, reflects the severity of depressive symptoms.

### 2.8. Statistical Analysis

In the descriptive analyses, we report on a variety of factors. These include sociodemographic characteristics, perinatal data, maternal risk factors, indicators of stress at birth, and measures of postnatal intimate mother–child contact. Each of these factors is stratified according to GA. To analyse these data, we employed specific statistical methods. Calculation of *p*-values was conducted using 1-way ANOVA for continuous variables and Pearson’s Chi-square tests for categorical variables. Reported *p*-values corresponded to two-tailed tests with a significance level set at α = 0.05. Further, recalled maternal rearing behaviour of the study participants stratified by GA and their mothers as well as correlations between recalled maternal rearing behaviour of the participants and their mothers were reported. Multiple linear regression analyses with different dimensions of recalled maternal rearing behaviour as outcomes were executed. In the first step, associations with GA (weeks of prematurity) and sociodemographic adjustment variables (age and gender) were assessed. The next step included perinatal stress indicators (birth weight percentile, Apgar score, perinatal adverse events, and time spent in the ICU), and then intimate mother–child contact (rooming-in, breastfeeding, and skin-to-skin contact) variables were added to the model. Applying this hierarchical procedure allows for a statistical test of whether its addition implicates statistically significant gains in explained variance (after accounting for all other variables that are included in the model) [47]. Lastly, discrepancy scores in recalled maternal rearing behaviour between subjects born preterm and at term and their mothers were calculated. Multiple linear regression analyses using discrepancy scores as the main predictor and controlling for GA, age, gender, and perinatal indicators were applied to test associations with depressive symptoms.

All effect sizes and regression coefficients were interpreted according to Cohen [48]. Analyses were performed using SPSS version IBM SPSS 20.0; SPSS, Inc., Chicago, IL, USA; R, version 4.0.0, for Windows. Collinearity was analysed with a correlation matrix.

## 3. Results

### 3.1. Sample Characteristics

The sample comprised 199 individuals (56.3% women; *n* = 112) with a mean age of 27.5 years (*SD* = 7.6). Regarding sociodemographic factors, there were differences between age groups in accordance with SES. SES and age were highest in the term-born (GA ≥ 37 weeks) group, with no difference observed between the groups regarding the sum score of depression (Table 1).

Individuals born preterm had a lower birth weight and more often a birth weight below 1500 g, and individuals born very preterm (before 32 weeks) had a birth weight below 1000 g (Table 1). Furthermore, significant differences between individuals born preterm and individuals born at term were found concerning postnatal indicators, such as higher maximum partial pressure of carbon dioxide (pCO_2_) and lower Apgar score for individuals born preterm. Individuals born preterm also experienced more perinatal adverse events. The number of days spent in the incubator increased with a lower GA, as well as the days spent in the ICU and the hospital.

The age of mothers at birth did not differ between the different GA groups (Table 1). The proportion of mothers smoking and consuming alcohol during pregnancy was relatively low but descriptively was the highest among the group of individuals born extremely preterm. Furthermore, mothers of individuals born preterm reported an emergency atmosphere and feelings of anxiety of losing control at the time of birth.

The contact between mother and child after birth differed between mothers of children born preterm and mothers of children born at term, whereby mothers of children born preterm had the first skin-to-skin contact later after birth than mothers of children born at term (Table 1). Furthermore, rooming-in was less likely for children born preterm. The proportion of breastfeeding was descriptively the lowest in the group with individuals born extremely preterm.

### 3.2. Recalled Maternal Rearing Behaviour from the Perspective of Individuals Born Preterm and at Term and Their Mothers 

The mean values of the separate dimensions of recalled maternal rearing behaviour were similar between individuals in this study and their mothers. Although small and insignificant differences were observed in specific GA groups, there were no significant differences between the different GA groups and any dimension of recalled maternal rearing behaviour (emotional warmth, rejection/punishment, control/overprotection, ambition, overprotection) (see Table 2).

When examining correlations between the recalled maternal rearing behaviour of individuals and that of their mothers, there were medium weak-to-weak significant correlations for all dimensions of recalled rearing behaviour, with the largest correlations for control/overprotection (*r* = 0.350, *p* < 0.001) and overprotection (*r* = 0.345, *p* < 0.001). Correlations were strongest for rejection/punishment (*r* = 0.228, *p* < 0.01) and ambition (*r* = 0.209, *p* < 0.01). Lastly, the correlation for emotional warmth from the perspective of individuals born preterm and term and respective mothers was the smallest (*r* = 0.174, *p* < 0.05).

### 3.3. Associations between Prematurity and Recalled Maternal Behaviour from the Perspective of the Child

Linear regression analyses revealed that the number of weeks of prematurity was associated with recalled maternal control/overprotection after controlling for sociodemographic features, perinatal stress indicators, and mother–child contact after birth (Table 3). Children born preterm with lower GA more often recalled their mothers as controlling and overprotective (B = 0.073, *p* = 0.045). There was a pattern for ambition that did not reach statistical significance (*p* = 0.065). There were no differences between individuals born preterm and at term for recalled maternal emotional warmth (*p* = 0.256), rejection/punishment (*p* = 0.968), and overprotection (individual category) (*p* = 0.206). It is important to highlight that the relationships between prematurity and recalled maternal rearing behaviour, while significant in some aspects, generally displayed medium weak-to-weak associations.

Regarding mother–child contact after birth, we observed associations between first-time skin-to-skin contact (kangaroo care) and recalled maternal rearing behaviour. Individuals who waited more days before the first-time skin-to-skin contact occurred reported more recalled maternal rejection/punishment (B = 0.071, *p* < 0.01), less recalled emotional warmth (B = −0.123, *p* < 0.05), and more control and overprotection (B = 0.119, *p* < 0.05). No associations between rooming-in and breastfeeding with recalled maternal rearing behaviour were found. 

Perinatal data were not associated with recalled maternal rearing behaviour, with no associations between birth weight, Apgar score, perinatal adverse events (yes/no), and recalled maternal rearing behaviour. There were gender differences, as female participants born preterm and at term recalled more emotional warmth, less control/overprotection, and less ambition from their mother compared with male preterm and term-born individuals.

### 3.4. Associations between Discrepancies in Perspectives on Recalled Maternal Rearing Behaviour between Individuals and Their Mothers and Depressive Symptoms

After controlling for prematurity, age, gender, and perinatal factors (birth weight percentiles, APGAR score, perinatal adverse events, duration of ICU stay), those who differed from their mothers in their perspective on maternal rejection/punishment reported significantly more depressive symptoms (B = 1.786, *p* < 0.05). No statistically significant associations were observed for the other dimensions of recalled maternal rearing behaviour, although non-statistically significant patterns could be seen for emotional warmth (*p* = 0.077) and ambition (*p* = 0.083). Prematurity, measured in weeks of GA, did not significantly predict depressive symptoms when the discrepancy in recalled maternal rearing behaviour and other confounders were included (for details, see Table 4).

## 4. Discussion

This study investigated recalled maternal rearing behaviour from the perspective of adult individuals born preterm and at term and their mothers. We examined whether early intimate mother–child contact (skin-to-skin contact, breastfeeding, and proximity at night/rooming-in) played a role in recalled maternal rearing behaviour decades later, and whether discrepancies in recalled maternal rearing behaviour between individuals and their mothers were associated with depressive symptoms in adulthood.

Our results reveal similarities between reported recalled maternal rearing behaviour of individuals born preterm and at term and their mothers within all dimensions of rearing behaviour. The correlations ranged from *r* = 0.174 for emotional warmth to *r* = 0.350 for control and overprotection, suggesting comparatively higher agreement regarding the facets of parental behaviour that are often perceived as more “negative” or related to conflictual issues in the parent–child relationship. Children born preterm with lower GA more often recalled their mothers as controlling and overprotective, which is in line with a meta-analysis including 27 independent data sets on individuals born preterm and reports of parenting styles of children born preterm being more controlling compared with parents of children born at term [12]. The finding that mothers reported a different emotional atmosphere (characterised by feelings of a loss of control and emergency) surrounding a preterm child’s birth holds the potential to understand these differences from the mother’s point of view. Studies show that, even during pregnancy, women who later give birth to a premature baby have higher anxiety and stress levels than mothers of full-term babies. Both psychological parameters are therefore considered risk factors for a preterm birth [49,50]. Previous studies have shown parenting behaviour is highly dependent on the parent’s mental health; for instance, mothers who reported higher levels of anxiety also displayed more controlling behaviours [51]. Furthermore, the premature infant itself suffers from physiological stress due to dysregulation of stress hormones such as cortisol, which can negatively affect long-term health [52]. This dysregulation could also impact the mother–child bond, since a higher cortisol response in children is linked to increased behavioural issues [53].

Empirical evidence, albeit from contexts other than perinatal stress, physical illness, and/or vulnerability, has highlighted that a parent’s and child’s characteristics interact in the emergence of specific behaviour/parenting styles [54]. This may be of particular importance in the research and clinical/practical support of families experiencing preterm birth and has previously been recognised from a conceptual standpoint [55]. More specifically, relating to the observed differences in recalled rearing behaviour, efforts to regain control through a more controlling parental rearing behaviour might be an understandable way to cope with an immensely stressful situation perceived as traumatic [56], the outcome of which is out of one’s hands. Such reactions to perceived uncontrollability have previously been studied in other medical contexts, mostly relating to chronic illness [57]. Evidence suggests that controlling and overprotective parental behaviour might be motivated by the desire to keep the child, who is perceived as vulnerable, safe and close to home [58]. This protective instinct, while understandable, underscores the delicate balance required in parenting practices, especially in the context of preterm birth. However, the transition from protection to control raises critical questions about the long-term impact on child development. A protective parenting style, which is expressed through structures and rules, could have a positive influence on the child [59], while, regarding future child development, a controlling rearing style could confer a higher risk of detrimental outcomes. Meta-analyses have shown controlling rearing behaviour is associated with externalising problems in childhood and adolescence (i.e., negative associations for behavioural control and positive associations for harsh and psychological control) [60] and academic achievement (i.e., positive associations for behavioural control and negative associations for harsh and psychological control) [61]. Therefore, screening parents of preterm children for distress and offering support could be helpful to counteract overly strict and limiting parental behaviour that is primarily driven by parents’ insecurities and has the potential to jeopardise the parent–child relationship and the child’s development of autonomy in the long run.

Possibly, not the rearing style but incongruence in recalled maternal rearing style between individuals and their mothers as a surrogate marker for differing rearing perception affects mental health. Although, in this study, we found a comparability in recalled maternal rearing style between participants born preterm and at term and their mothers, small discrepancies affected the mental health of the individuals born preterm and at term. Incongruence in recalled maternal rearing style co-occurred with more depressive symptoms, especially in the case of rejection/punishment. Non-significant patterns could be seen for emotional warmth (*p* = 0.077) and ambition (*p* = 0.083) but not gestational age.

Regarding mother–child contact after birth, our results revealed that individuals who waited longer before the first skin-to-skin contact occurred reported more recalled maternal rejection/punishment, less recalled emotional warmth, and more control and overprotection. These findings suggest that early affective contact could set the course for a better or worse parent–child relationship, mirroring findings of previous studies reporting the mother–infant bonding phenomenon that adds a dimension of responsibility and competence to the new mother [24,62]. They are in line with meta-analyses that have shown concordances between early and late bonding experiences throughout development and identified preterm birth as a risk factor for the quality/opportunities for early bonding experiences [63]. Furthermore, a study following children until the age of ten reported more attachment behaviour and greater mother–child reciprocity when early (immediately after birth) skin-to-skin care occurred [64]. These findings underscore the importance of skin-to-skin contact. Nevertheless, barriers to its implementation in clinical practice persist to this day. A study conducted in neonatal intensive care units across Italy revealed that, while skin-to skin contact is offered as standard practice, the duration varies significantly among hospitals. Furthermore, evidence suggests that documenting kangaroo care leads to a notable enhancement [65]. A review from 2023 recommends providing skin-to-skin contact for premature or low-birth-weight infants for a minimum of 8 h per day [66]. However, recommendations in this regard vary considerably and warrant further investigation. This study emphasises the importance of immediate mother–child contact after birth for the life course of newborns. Clinicians should document data regarding skin-to-skin contact, including the duration in days and the “dosage” in hours or sessions of skin-to-skin contact in medical records. This practice would not only enhance the scope of skin-to-skin contact but also enable more accurate assessments and recommendations.

### Strengths and Limitations

This study is the first to comprehensively record the circumstances of preterm birth, including the clinical course thereafter, and examine subjects born preterm an average of three decades after birth. Studies often lack this additional information on recalled maternal rearing style from the perspectives of the already older mothers and data on the intimate mother–child contact immediately after birth reported by these mothers and from medical records. Furthermore, recalled parental rearing behaviour was assessed via self-report and reflects the inner perspective on rearing style from the view of the study participants and their mothers, but no comparison with actual rearing behaviour could be made. Due to the strong correlations of reported recalled maternal rearing behaviour between subjects born preterm and at term and their mothers, one can assume rearing behaviour is in line with recalled rearing style. Lastly, there was no information on life events or life courses in general and therefore could not be included in the analyses. During the present analysis, there were examinations of specific patterns that were considered most relevant to the present research questions. Nevertheless, the potential for further significant associations between the different parameters used in the present study must be acknowledged. However, it is important to realize that the complexity of the data might hold further insights than those presented here. Future research could explore these relationships further and potentially uncover additional significant associations that could contribute to a more comprehensive understanding of the topic.

## 5. Conclusions

This study contributes to the understanding of preterm birth and recalled maternal rearing behaviour, underscoring the relevance of preterm earliest and closest social relationships and stressing the importance of close intimate contact between mother and child immediately after birth and its sustained relevance decades after birth. It appears that different levels of premature birth influence the parenting style of the mother and that the waiting time for first skin contact with the mother is longer in premature born individuals compared with individuals born at term, which also affects the range of feelings later remembered by the son/daughter.

## Figures and Tables

**Table 1:** Sample characteristics of subjects born preterm and at term including data on birth, postnatal course, and intimate mother–child contact after birth.

	All (*N* = 199)	Group 1: GA ≥ 37 (*n* = 64)	Group 2: GA 33–36 (*n* = 61)	Group 3: GA 29–32 (*n* = 47)	Group 4: GA ≤ 28 (*n* = 27)	*p*
Sociodemographic Factors						
Sex						0.137
men	87 (43.7%)	28 (43.8%)	20 (32.8%)	24 (51.1%)	15 (55.6%)	
women	112 (56.3%)	36 (56.3%)	41 (67.2%)	23 (48.9%)	12 (44.4%)	
Age	27.5 ± 7.6	30.1 ± 8.5	26.4 ± 6.9	26.8 ± 7.4	25.1 ± 5.2	0.007
SES	12.5 ± 3.6	13.9 ± 3.4	11.9 ± 3.7	12.4 ± 3.4	10.6 ± 3.1	0.000
SES groups						0.006
low	36 (18.1%)	4 (6.3%)	14 (23.0%)	9 (19.1%)	9 (33.3%)	
medium	112 (56.3%)	35 (54.7%)	33 (54.1%)	28 (59.6%)	16 (59.3%)	
high	51 (25.6%)	25 (39.1%)	14 (23.0%)	10 (21.3%)	2 (7.4%)	
Working status						0.129
employed/self-employed	114 (57.3%)	43 (67.2%)	30 (49.2%)	26 (55.3%)	15 (55.6%)	
unemployed	12 (6.0%)	3 (4.7%)	2 (3.3%)	3 (6.4%)	4 (14.8%)	
student/trainee	73 (36.7%)	18 (28.1%)	29 (47.5%)	18 (38.3%)	8 (29.6%)	
Family status						0.115
married	34 (16.9%)	17 (26.6%)	11 (18.0%)	6 (12.8%)	0 (0%)	
Single	165 (83.1%)	47 (73.4%)	50 (82.0%)	41 (87.2%)	27 (100%)	0.002
Depressive symptoms	4.0 ± 4.4	4.2 ± 4.2	3.7 ± 3.7	3.8 ± 4.1	4.6 ± 6.5	0.703
Birth weight data						
Birth weight (kg)	2.193 ± 1.013	3.450 ± 0.378	1.974 ± 0.459	1.532 ± 0.411	0.859 ± 0.224	0.000
Birth weight < 1500 g	57 (28.6%)	0 (0%)	10 (16.4%)	20 (42.6%)	27 (100%)	0.000
Birth weight < 1000 g	25 (12.6%)	0 (0%)	0 (0%)	6 (12.8%)	19 (70.4%)	0.000
Birth weight percentiles	37.57 ± 26.26	46.86 ± 23.23	19.97 ± 20.55	48.02 ± 27.29	37.11 ± 22.71	0.000
Maternal risk factors						
Age at birth	31.53 ± 4.59	31.89 ± 4.80	30.72 ± 4.25	31.74 ± 4.82	32.19 ± 4.35	0.402
Smoking during pregnancy (yes)	15 (7.5%)	5 (7.8%)	4 (6.6%)	3 (6.4%)	3 (11.1%)	0.879
Alcohol during pregnancy (yes)	5 (2.5%)	1 (1.6%)	1 (1.6%)	1 (2.1%)	2 (7.4%)	0.377
Postnatal indicators						
Perinatal adverse events (yes)	19 (9.5%)	1 (1.6%)	1 (1.6%)	4 (8.5%)	13 (48.1%)	0.000
pH values	7.28 ± 0.10	7.27 ± 0.07	7.29 ± 0.08	7.27 ± 0.14	7.26 ± 0.14	0.473
Intubation (yes)	61 (30.7%)	1 (1.6%)	10 (16.4%)	24 (51.1%)	26 (96.3%)	0.000
Intubation days	4.82 ± 11.86	0.02 ± 0.13	0.25 ± 0.65	6.49 ± 9.87	23.67 ± 20.45	0.000
Apgar score 5 min	8.39 ± 1.66	9.56 ± 0.77	8.49 ± 1.36	7.60 ± 1.58	6.78 ± 1.91	0.000
pCO_2_ max (mmHg)	56.00 ± 14.52	49.76 ± 11.75	51.41 ± 11.64	65.22 ± 15.05	65.10 ± 13.14	0.000
Days incubator	16.61 ± 23.36	0.00 ± 0.00	9.65 ± 12.76	23.14 ± 17.27	60.35 ± 19.87	0.000
Days ICU	17.99 ± 30.08	0.43 ± 2.39	4.13 ± 7.30	28.30 ± 30.26	72.96 ± 24.38	0.000
Days hospital stay (days)	45.07 ± 43.49	7.28 ± 4.10	33.16 ± 18.42	71.62 ± 33.95	115.30 ± 36.05	0.000
Stress indicators birth						
Emergency atmosphere (yes)	101 (51.8%)	5 (7.9%)	34 (56.7%)	36 (80.0%)	26 (96.3%)	0.000
Anxiety of losing control (yes)	38 (9.7%)	2 (3.1%)	15 (25.9%)	9 (20.0%)	12 (46.2%)	0.000
Postnatal mother–child contact						
Breastfeeding (yes)	118 (59.3%)	41 (64.1%)	36 (59.0%)	28 (59.6%)	13 (48.1%)	0.573
First-time skin-to-skin contact (days)	1.89 ± 2.68	0.20 ± 0.29	1.69 ± 1.74	3.03 ± 3.41	4.28 ± 3.34	0.000
Rooming-in (days)	0.26 ± 0.72	0.53 ± 0.95	0.23 ± 0.72	0.02 ± 0.15	0.00 ± 0.00	0.000

Note. Categorical variables are reported as number and percentage and continuous variables as mean and standard deviation. Calculation of *p*-values by 1-way ANOVA for continuous variables and Pearson’s Chi-square for categorical variables; reported *p*-values correspond to two-tailed tests (significance level: α = 0.05). Abbreviations: SES—socioeconomic status (3–21, with higher scores indicating better socioeconomic status); GA—gestational age; ICU—intensive care unit. Perinatal adverse events: occurrence of intraventricular haemorrhage, occurrence of necrotising enterocolitis, and moderate or severe bronchopulmonary dysplasia were summarized as adverse events. First-time skin-to-skin contact refers to so-called kangaroo care; rooming-in refers to sleeping in the same room as the child during the hospital stay.

**Table 2:** Recalled maternal rearing behaviour from the perspectives of subjects born preterm and at term and their mothers.

	All (*N* = 199)	Group 1: GA ≥ 37 (*n* = 64)	Group 2: GA 33–36 (*n* = 61)	Group 3: GA 29–32 (*n* = 47)	Group 4: GA ≤ 28 (*n* = 27)	*p*
Emotional Warmth						
Subject	6.57 ± 1.34	6.60 ± 1.12	6.77 ± 1.27	6.38 ± 1.43	6.41 ± 1.74	0.539
Mother	6.56 ± 1.03	6.47 ± 0.99	6.69 ± 1.03	6.55 ± 1.16	6.52 ± 0.89	0.671
Rejection/Punishment						
Subject	2.21 ± 0.53	2.22 ± 0.42	2.14 ± 0.35	2.22 ± 0.47	2.30 ± 0.99	0.664
Mother	2.18 ± 0.44	2.19 ± 0.43	2.16 ± 0.49	2.19 ± 0.45	2.15 ± 0.36	0.922
Control/Overprotection						
Subject	3.33 ± 1.23	3.35 ± 1.22	3.45 ± 1.20	3.25 ± 1.08	3.15 ± 1.56	0.367
Mother	3.50 ± 1.14	3.34 ± 0.95	3.44 ± 1.13	3.57 ± 1.25	3.89 ± 1.31	0.405
Ambition						
Subject	1.57 ± 0.77	1.71 ± 0.83	1.47 ± 0.66	1.51 ± 0.66	1.56 ± 0.97	0.309
Mother	1.44 ± 0.56	1.44 ± 0.53	1.39 ± 0.53	1.51 ± 0.62	1.41 ± 0.57	0.813
Overprotection						
Subject	1.75 ± 0.84	1.63 ± 0.79	1.98 ± 0.91	1.73 ± 0.79	1.59 ± 0.84	0.069
Mother	2.07 ± 0.87	1.91 ± 0.71	2.05 ± 0.92	2.06 ± 0.87	2.48 ± 0.98	0.097

Note: Data are reported as mean values with standard deviations; *subject* refers to preterm and term-born individuals at the UMCM who reached adulthood at the time of this study; *p*-value calculated by independent samples and Kruskal–Wallis test.

**Table 3:** Associations of prematurity and intimate mother–child contact after birth on recalled maternal rearing behaviour decades later.

	Rejection/ Punishment			Emotional Warmth			Control/ Overprotection			Ambition			Overprotection		
	*B* (*SE*)	*β*	*p*	*B* (*SE*)	*β*	*p*	*B* (*SE*)	*β*	*p*	*B* (*SE*)	*β*	*p*	*B* (*SE*)	*β*	*p*
Prematurity (weeks)	0.001 (0.015)	0.005	0.968	−0.042 (0.037)	−0.149	0.256	0.073 (0.036)	**0.277**	**0.045**	0.041 (0.022)	0.252	0.065	0.031 (0.025)	0.178	0.206
Age (years)	0.003 (0.006)	0.050	0.561	−0.018 (0.014)	−0.104	0.216	−0.022 (0.014)	−0.141	0.112	−0.006 (0.009)	−0.056	0.516	−0.018 (0.010)	−0.169	0.063
Gender (women)	0.074 (0.082)	0.067	0.369	**0.638 (0.200)**	**0.234**	**0.002**	**−0.492 (0.195)**	**−0.195**	**0.012**	**−0.424 (0.119)**	**−0.271**	**0.000**	−0.080 (0.133)	−0.047	0.549
BW percentiles	0.000 (0.002)	0.001	0.993	−0.001 (0.004)	−0.013	0.861	−0.002 (0.004)	−0.040	0.602	−0.001 (0.002)	−0.019	0.796	−0.001 (0.002)	−0.040	0.609
Apgar score	0.052 (0.032)	0.156	0.103	0.039 (0.077)	0.048	0.611	0.084 (0.075)	0.110	0.266	0.041 (0.046)	0.087	0.371	0.044 (0.051)	0.087	0.387
Perinatal adverse events (yes)	0.314 (0.174)	0.170	0.072	0.144 (0.418)	0.032	0.732	−0.506 (0.408)	−0.121	0.216	−0.418 (0.250)	−0.160	0.096	−0.083 (0.280)	−0.030	0.767
Duration of ICU stay (days)	−0.002 (0.002)	−0.089	0.468	−0.005 (0.005)	−0.112	0.351	0.008 (0.005)	0.192	0.131	0.005 (0.003)	0.189	0.131	0.003 (0.004)	0.109	0.399
Breastfeeding (yes)	0.008 (0.084)	0.007	0.924	0.196 (0.204)	0.071	0.338	0.049 (0.199)	0.019	0.806	0.021 (0.121)	0.013	0.862	0.013 (0.136)	0.008	0.925
Skin-to-skin contact (days)	**0.071 (0.021)**	**0.346**	**0.001**	**−0.123 (0.050)**	**−0.246**	**0.015**	**0.119 (0.049)**	**0.255**	**0.017**	0.059 (0.030)	0.203	0.053	0.059 (0.034)	0.190	0.080
Rooming-in (days)	0.051 (0.059)	0.067	0.390	0.106 (0.142)	0.057	0.455	−0.115 (0.138)	−0.067	0.407	−0.054 (0.084)	−0.051	0.520	−0.059 (0.095)	−0.051	0.536

Note: Coding of predictors. Bold numbers are meant to highlight significant outcomes. Weeks of prematurity: continuous (40/gestational age); age: continuous (years); gender: 0 = men, 1 = women; BW percentiles: continuous; Apgar score: continuous (Apgar score after 5 min); perinatal adverse events: 0 = no, 1 = yes; perinatal adverse events: occurrence of intraventricular haemorrhage and/or occurrence of necrotising enterocolitis and/or moderate or severe bronchopulmonary dysplasia were summarized as adverse events; duration of ICU stay: continuous (days); breastfeeding: 0 = no, 1 = yes; skin-to-skin contact represents first-time skin-to-skin contact (so-called kangaroo care): continuous (days); rooming-in (sleeping in the same room as the child during the hospital stay): continuous (days). Recalled maternal rejection/punishment: adj. R^2^ = 0.081; recalled maternal emotional warmth: adj. R^2^ = 0.125; recalled maternal control/overprotection: adj. R^2^ = 0.030; recalled maternal ambition: adj. R^2^ = 0.054; recalled maternal overprotection: adj. R^2^ = −0.020.

**Table 4:** Associations between discrepancies in recalled maternal rearing behaviour between subjects born preterm and at term and their mothers and depressive symptoms.

	Depression			
	*B* (*SE*)	*β*	*p*	Adjusted R-Squared
Discrepancy in recalled maternal				
Rejection/punishment	1.786 (0.743)	0.169	0.017	0.123
Emotional warmth	1.189 (0.668)	0.124	0.077	0.118
Control/overprotection	0.779 (0.661)	0.084	0.240	0.101
Ambition	1.077 (0.617)	0.121	0.083	0.109
Control	0.899 (0.643)	0.100	0.164	0.104

Note: In all models, the following confounders were included: weeks of prematurity, age, gender, birth weight percentiles, Apgar score, perinatal adverse events, and duration of ICU stay. Weeks of prematurity was not a significant predictor of depression symptoms in any of the models.

## Data Availability

A.F. had full access to all the study data and had responsibility for the integrity of the data and the accuracy of the data analysis. Statistical analyses were performed by A.F. The analysis presents clinical data and constitutes a major scientific effort with high methodological standards and detailed guidelines for analysis and publication to ensure scientific analyses are of the highest level. Therefore, data are not made available for the scientific community outside the established and controlled workflows and algorithms. To meet the requirements of verification and reproducibility of scientific findings, we offer access to the local database upon request at any time. Interested researchers should make their requests to the coordinating PI (Achim Fieß; achim.fiess@unimedizin-mainz.de). More detailed contact information is available at the UM homepage (www.unimedizin-mainz.de).
